# Anti-Infective, Anti-Inflammatory, and Immunomodulatory Properties of Breast Milk Factors for the Protection of Infants in the Pandemic From COVID-19

**DOI:** 10.3389/fpubh.2020.589736

**Published:** 2021-03-02

**Authors:** Pasqua Anna Quitadamo, Laura Comegna, Pierpaolo Cristalli

**Affiliations:** NICU “Casa Sollievo della Sofferenza” Foundation, Scientific Research and Care Institute, San Giovanni Rotondo, Italy

**Keywords:** antiviral activity, functions human milk factors, COVID 19, clinical application of human milk factors, human milk research

## Abstract

COVID-19 pandemic since the end of 2019 spreads worldwide, counting millions of victims. The viral invasion, systemic inflammation, and consequent organ failure are the gravest features of coronavirus disease 2019 (COVID-19), and they are associated with a high mortality rate. The aim of this study is to evaluate the role of breast milk in the COVID-19 pandemic, analyzing its antiviral, anti-inflammatory, and immunoregulatory effects due to its bioactive components, so numerous and important for the protection of infants. The study tried to demonstrate that all the components of human milk are capable of performing functions on all the pathogenic events recognized and described in COVID-19 disease. Those human milk factors are well-tolerated and practically free of side effects, so breast milk should become a research topic to discover therapies even in this epidemic. In the first part, the mechanisms of protection and defense of the breast milk elements will be delineated; in the second section, it will describe the human milk effects in viral infections and it will be hypothesized how the known mechanisms could act in COVID infection.

## Highlights

- To protect infants and newborns from COVID-19, they would be fed breast milk. Where mother's milk is not available, or for fragile babies (premature or newborns with pathologies), the alternative must be represented by human milk donated to the Human Milk Banks, which, although pasteurized, retains most of its anti-infectious properties (e.g., total savings of human milk oligosaccharides and fatty acids). Whenever human milk substitutes are needed, formulas added to milk bioactive factors should be preferred.
- Human milk factors are well-tolerated and practically free of side effects, so breast milk should become a research topic aimed to identify therapies even in this epidemic.
- Many components of human milk may have a potential therapeutic activity, and they are under evaluation in infections, inflammatory bowel diseases, hypertension, cognitive decline, and cancer. Most of the bioactive factors of human milk may interact synergistically with each other or with the immune response. During this study, we tried to demonstrate that human milk mediators might have a function in all events recognized and described in COVID-19.

## Introduction

Since the end of 2019, an acute severe viral infection has emerged with rapid transmission around the world to over a million people within a few months. Named coronavirus disease 2019 (COVID-19) by the World Health Organization, the pandemic disease has resulted in one of the largest global public health problems in modern history. Severe acute respiratory syndrome coronavirus 2 (SARS-CoV-2) infects cells in the respiratory system and causes inflammation and cell death. Subsequently, the virus spreads out and damages other vital organs and tissues, triggering a complicated spectrum of pathophysiological changes and symptoms. Systemic inflammation and consequent respiratory, circulatory, and renal failure are the severe features of COVID-19 and are associated with a very high mortality rate. Given the lack of vaccine and antiviral therapy and the non-existent herd immunity, there is an urgent need to find therapeutic solutions to stop the spread of the infection and to effectively treat the disease; this is the highest priority among health care providers, government authorities, and pharmaceutical industry.

For more than 20 years, the protective power of breast milk has been known against many diseases and with special regard to infections due to its richness in immune-related factors like human milk oligosaccharides, milk proteins and lipids, and other active mediators (see section Breast Milk and Mediators and Breast Milk and Viral Infection).

Undoubtedly, this breast milk power can be ascribed to the large number of bioactive molecules that have been proven to be protective against infections, bringing down inflammation, facilitating immune system and organ growth, and influencing the infant microbiome, at a developmental stage where the infant's own immune system is relatively immature and naive (see sections Breast Milk and Mediators and Breast Milk and Viral Infection).

Nowadays, with the increased phenomenon of antibiotic resistance, national and international research has the purpose to discover new antibiotics or natural substances that can act as antibiotics. Breast milk has become the subject of many of these researches and on others about inflammatory diseases, cancer, and other pathologies. Preclinical investigations on human milk are translating into clinical applications, up to the large-scale production of the active molecules derived from it (1).

The aim of this study is to evaluate the role of breast milk in the COVID-19 pandemic, analyzing its antiviral, anti-inflammatory, and immunoregulatory effects due to the bioactive components. Few data are available about the human milk's potential role against COVID-19, but a lot of studies documented its antiviral effect against many viruses. The following is a brief recap of the current evidence on the mechanisms that explain its multiple functions in the context of anti-infective responses to deduce the possible practical application of breast milk against this pandemic.

In the first part, the known mechanisms of protection and defense of the individual elements contained in breast milk will be delineated (Table 1), while in the second section, human milk effects in viral infections will be described.

**Table 1:** Functions of breast milk factors.

	**Antiviral**	**Antinflammatory**	**Immunomodulation**	**Anticoagulant**	**Repair of the damage**
HMO >20 g/L in colostrum 12 g/L in mature milk	Direct: inhibiting the adhesion of pathogens Indirect: act as a soluble analog of the receptor for pathogens, prebiotics	Prevent and decrease inflammatory response Inhibit interferon production Suppress activation of the NF-kB Inhibit the adhesion of leukocytes to the endothelium triggering inflammatory pathway Suppress the infiltration of leukocytes Decrease acute phase inflammatory cytokine secretion Promote healthy intestinal microbiota Regulate commensal bacteria with antinflammatory response	Promote the maturation of the immune system Create a more balanced Th1/Th2 cytokine response Modulate immune signaling pathways, including TLR3, TLR5, PAMP		Stimulate maturation of epithelial cells
Cytokines					
IL1 β		Attenuates the activation of pro-inflammatory IL-8 (1.2 ng/L**)**	Suppresses proinflammatory responses of NF-kB signaling		
IL-1RA IL-1 receptor antagonist		Competes with the pro-inflammatory cytokine, IL-1, for receptor binding			
IL-2			Recruits T cell to stimulate an antigen-specific immune response Play an important role in the differentiation of neonatal T cells toward a preferential Th1, rather than Th2		Stimulate the proliferation and differentiation of the former and greater vitality in the latter
IL 6 1.6 ng/L		Has pro-inflammatory properties and is present in the acute phase of infection			
IL 10 0.13 ng/L		Inhibit the activity of Th1 effector cells, NK cells and macrophages and the production of several pro inflammatory cytokines (IL-1, IL-6, IL-8, and TNF-α)	Provide immunoregulation Attenue the immune response Promote immunoglobulin synthesis		
TGFβ 1.5 mg/ml		Decreas pro-inflammatory cytokine expression.	Inhibits naïve T cells from differentiation into Th1 and Th2 subtypes Stimulates intestinal defense by switching immunoglobulin classes from IgM to IgA in B lymphocytes		Assistance with intestinal mucosal repair
TNFα 620 ± 183 pg/mL		In association with its soluble receptor riduce its pro-inflammatory activity			
EGF 200 μg/l in colostrum 30–50 μg/l in mature milk		Upregulation of IL 10 Decrease livel of IL18 and mRNA IL18			Promote growth and maturation of the fetal pulmonary epithelium Stimulates synthesis of DNA in the digestive tract Acelerates wound healing Role in repair of the intestine during intestinal injury or infection
HB-EGF concentrations 2 to 3 times lower than EGF	Bind to pathogen	Decrease pro-inflammatory citokine expression			
VEGF 74.3 ± 34.9 ng/ml on the first day 6.2 ± 2.3 ng/ml on the fifth day 4 ng/ml in mature milk.		Riduce TNFα and IL-6 levels			Stimulate angiogenesis Prevent intestinal edema
Chemochine G-CSF MIF			Factor inhibitory macrophage migration		Intestinal trophic factor
Adipokins		Influence polarization of macrophages to anti-inflammatory phenotype	Stimulate t cell T-lymphocyte response Regulate immune response and prevent inflammation		Role in fluid homeostasis and regulation of cardiovascular system Angiogenesis
Lipids omega-3 PUFAs Omega-3 + omega-6 PUFAs Free fatty acids (FFAs) and monoglycerides EPA Oxysterols	Inactivate pathogens Lytic effect on several viruses Inhibit the viral replication	Inhibit expression of adhesion molecules Downregulate proinflammator gene Decrease NF-kB, bind to PPAR-γ Antiossidants properties Increase anti-inflammatory microbes, such as Lactobacillus and Bifidobacterium species Antagonize the pro-inflammatory effect of AA	Inhibits T-cell response (modification of lipid rafts and caveolae structure of membrane) Decrease degree of inflammator response-change membrane PL concentration Decrease platelet activating factor (PAF) induced TLR4 activation inhibit leukocyte migration Involved in the innate immune response against viruses	Antithrombotic properties less thrombin production -less time of clot destruction minor alterations of fibrinogen -reducing platelet aggregation by changing the composition of platelet membrane phosfoptides	Affect cell growth and apoptosis Maintaining membrane integrity Neurotrophic effect Stimulus to the production of neurons by stem cells
Casein and lactoalbumin	Inhibit the adherence to human respi- ratory tract epithelial cells Prevents the attachment to the mucosal acting as a receptor analog Inhibit the proteases of viruses				Alpha-lactalbumin induces apoptosis-like death with oleic acid
Secretory IgA 12 mg/ml in colostrum 1 mg/ml in mature milk	Bind to pathogens Inhibition of pathogen attachment to mucosal surfaces	Prevent inappropriate inflammatory responses to pathogenic microbes and antigens Influence intestinal microbiome	Decrease the activity of pattern recognition receptors Immune exclusion		
IgM 2–7 mg/100 ml IgG 1-3 mg/100 ml	High avidity IgM antibodies reactive with viruses bind, opsonization and agglutination of pathogens	Prevention of typical inflammatory response	Immune-surveillance		Inhibition of hydroxyl radical formation
Lysozyme 3–110 mg/100 ml	Inactivate, destroy or block the attack of specific microbes on the surfaces of the mucous membranes	Eliminate trigger for acute inflammatory response			
Lattoferrin (lactoferricin) 0,5 gr/dl in colostrum, 0.2 gr/dl at 1 month, 0.1 gr/dl later	Iron sequestration Act as natural barrier of both respiratory and intestinal mucosa Revert the iron disorders related to the viral colonization LPS/lipothecoic acid interaction Serin protease activity Proteoglycans interaction and inhibition of replication of virus Cell wall breakdown	Eliminate trigger for acute inflammatoy response Inibit IL-1, IL-6, TNF-α, IL-8 Promote growth of probiotics	Decrease pro-inflammatory cytokine expression Block excessive DC activation upon TLR-induced inflammation Regulate intestinal microbiome Regulate innate and acquired immunity Enhancing the activation of immune cells	Anti-fibrinolytic property	Cell proliferation Maturation of gut enzymes
Lacthoaderin 3–33 mg/100 ml	Binds specifically virus Inhibits virus replication	Induces IL-10 and TGF-b release from regulatory T cells Enhances the ability of macrophages to phagocytize apoptotic cells Blocks NK-kB pathway via TRL4 inhibition	Prevent pro-inflammatory signaling and decrease inflammatory response Attenuate sepsis-induced apoptosis	Function as an anticoagulant and regulate haemostasis via homology of the C2 domain to coagulation factors VIII and V and the competition between them for PL membrane-binding sites	Inhibits excessive neutrophils infiltrating to organs Moderate attenuation of organ injury Improves the histopathology, the integrity of the lung tissue and decreases lung apoptosis Reduction of renal tissue histological damage Promotion of mucosal wound healing Attenuation of intestinal inflammation Facilitate neoangiogenesis
B defensin	Antimicrobial activity	Inhibit TLR7	Enhances TLR4/CD44–dependent intestinal epithelial defense		Decrease in the long-term risk of gastrointestinal inflammatory diseases
TLRs		Depress TLR signaling Decoy receptor to inhibit IL-8, TNF	Prevent pro-inflammatory cytokine expression and inflammation		
Chondroitin sulfate	Antiviral agent	Decrease production of proinflammatory cytokines Stimulate specific antioxidant enzymes	Inhibit activity of nuclear-factor kappalight-chain enhancer of activated B cells Promote the proliferation of anti- inflammatory IL-10–secreting T-regulatory cells	Has functions in coagulation	Enhance wound healing
Antioxidants		Scavange free radicals	Prevent inflammation		Prevent injury
Anti-proteasis (α-1-antitrypsin, α-1-antichymotrypsin, elastase inhibitor) α1-Antitrypsin 2–5 mg/100 ml		Metabolize protease produced by inflammatory cells	Prevent excessive inflammatory response		
MicroRNA 47,240 μg/L		Release of inflammatory mediators Proliferation of neutrophils and monocytes	Regulation of T- and B-cell development Regulation of function of dendritic cells and macrophages		Epigenetic regulation of stem cells fate and function
Cells 10 000 to 13 000 000 cells/ml		Inhibit NF-kB pathway Decrease pro-inflammatory cytokines and iNOS Increase SCFA production	Promote anti-inflammatory commensal bacteria proliferation Decrease inflammatory response Protective properties Ability to infiltrate the infant's tissue		Restore intestinal microbiome homeostasis Repair of tissue or organ damage Produce VEGF and HGF
Others Protectin PGE1/E2	Inhibition of MAC (membrane attack complex)	Inhibition of neutrophil enzyme, release, superoxide			Citoprotective effect

In the last part, it will be hypothesized how these mechanisms could counteract COVID-19 infection in its different stages starting from the knowledge that has currently been acquired about this new epidemic.

## Breast Milk and Mediators

The human milk interacts with the newborn's gut to increase the immune response against infection, and it can be considered like an immune system that includes many immunomodulatory mediators. It is known that human milk changes its immunological constituents related to the time from the delivery and maybe to the nutritional state of the mother. Recently, some authors suggest that there is modification also to response to the active needs of the nursing baby (2, 3). Riskin et al. (4) have shown in their study that the total number of white blood cells increases in human milk in relation to nursing infant's infection, specially the number of macrophages; so, the study underlines the immunological role of human milk.

The intake of immunomodulatory constituents of human milk confers protection against the incidence and severity of gastrointestinal and respiratory diseases, providing passive protection through innate and adaptive mother's immunity (5). It is known that breastfed infants are more protected compared with formula-fed infants (6). The human milk carries immunoglobulins, lactoferrin (LF), and oligosaccharides that provide passive protection and other mediators like pro- and anti-inflammatory factors [tumor necrosis factor-α (TNF-α), interleukin 1 (IL-1), prostaglandins E2 (PGE2), IL-10, transforming growth factor β (TGF–β), Thromboxane A2 (TXA2), Ribonucleid Acid Messenger RNA (mRNA)], mediators of leukocyte differentiation, and antibodies. The human milk lymphocyte pattern includes activated T cells and memory T cells more activated than lymphocyte pattern in peripheral blood (7). In addition, antibodiescarried by human milk protect infants against microorganisms; the prevalent antibodies are Secretory Immunoglobuline A (SIgA) produced by plasma cells in mammary glands that can neutralize microbial toxin bacteria and virus. Similarly, SIgM is important against enteric antigens (8, 9).

### Human Milk Oligosaccharides

Human milk oligosaccharides (HMOs) are a group of structurally unconjugated glycans, carbohydrates with a highly complex structure, from three to 10 monosaccharides, and differ in the degree of polymerization, carbohydrate content, type of glycosidic bonds, and ramifications (10). They are highly abundant and unique in human milk and represent the third largest solid component after lactose and lipids (11) (from 5 to 12 g/L in mature milk to >20 g/L in colostrum). More than 200 isomers and 162 chemical structures are known (10, 12, 13). HMOs are classified into three groups: neutral fucosylated (including 20-fucosyllactose, 20-FL), neutral non-fucosylated [including lacto-N-neotetraose (LNnT)], and acidic [including 30-sialyllactose (30-SL) and 60-sialyllactose (60-SL)] (14–16). Fucosylated HMOs are the most conspicuous component (77%), while sialylated HMOs represent 16%. HMOs are not a primary energy source for the child but perform important biological functions: enhance gut maturation; act as prebiotics, anti-adhesives, and antimicrobials; and directly modulate immune responses; thus, they may play an important role in the protective health effects of breast milk (17, 18). They are not digested, and they reach the systemic circulation intact (19, 20) so they may exert their functions.

HMO structure depends on two specific genes, and each lactating mother synthesizes a unique subset, influenced by genetics and enzymes that process HMOs (21, 22). Furthermore, in terms of quantity and structure, HMOs vary from woman to woman and in the same woman during breastfeeding (23).

### Functions of Human Milk Oligosaccharides

#### Interaction With Pathogens

**Figure 1:**
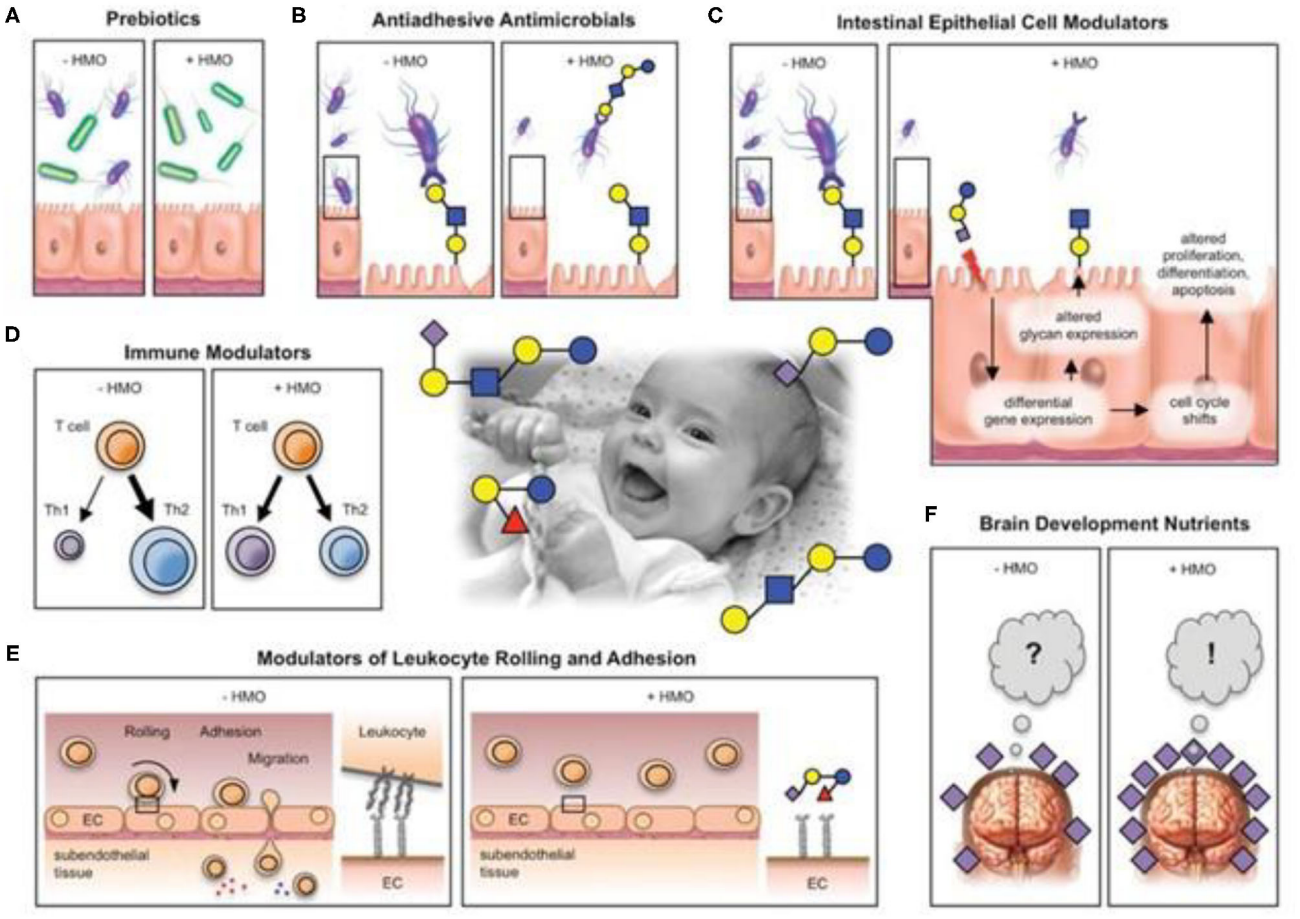
Postulated HMO effects. HMos may benefit breast-fed infant in multiple different ways. **(A)** HMOs are prebiotics that serve as metabolic substrates for beneficial bacteria (green) and provides them with a growth advantage over potential pathogens (purple). **(B)** HMOs are antiadhesive antimicrobials that serve as soluble glycan receptor decoys and prevent pathogen attachment. **(C)** HMOs directly affect epithelial cells and modulate their gene expression, which leads to changes in cell surface glycans and other cell responses. **(D)** HMOs modulate lymphocyte cytokine production leading to a more balances TH1/Th2 response. **(E)** HMOs reduce selectin-mediated cell-cell interactions in the immune system and decrease leukocyte rolling on activated endothelial cells, potentially leading to reduced mucosal leukocyte infiltration and activation. **(F)** HMOs provide Sia as potentially essential nutrients for brain development and cognition. (Center photo taken form author's personal collection). Bode (24).

HMOs are potent antimicrobial factors in human milk (Figure 1). Viral, bacterial, or protozoan pathogens need to adhere to mucosal surfaces to colonize or invade the host and cause disease. HMOs reduce infections by serving as anti-adhesive antimicrobials (25, 26), inhibiting the adhesion of pathogens to the surface of the host cells. Many HMOs act as a soluble analog of pathogen receptors, they have a structure very similar to cell surface receptors (glycocalyx) (anti-adhesion property), and they modify the expression of glycocalyx gene (modifying property) (11, 17). This mechanism modulates the expression of immune signaling genes, which have been shown to repress inflammation of the mucosal surface (27, 28). So, anti-adhesive activity and probiotic activity secondarily reduce inflammation within the intestine (29). HMOs and glycans also inhibit infection acidifying the gut lumen (28); they produce bacteriocins and organic acids that prevent pathogen growth (30).

Sialylated oligosaccharides can act as inhibitors against the enterotoxins produced by *Vibrio cholerae* and *Escherichia coli*. In mice infected with adherent-invasive *E. coli* (AIEC), 20-FL was effective in reducing pathogen colonization, modulating AIEC-induced CD14 expression and attenuating inflammation (31).

Physiological concentrations of HMOs have been able to inhibit the adhesion of *Entamoeba histolytica*, responsible for a widespread parasitic disease characterized by high mortality, to the layers of enteric epithelial cells and its cytotoxicity in a dose-dependent manner. The main adhesion-related virulence factor in *E. histolytica* is a lectin that binds with galactose and N-acetyl-galactosamine; HMOs contain terminal galactose, such as lacto-N-tetraose (LNT), that acts as decoy receptors. Glycan-mediated attachment mechanisms have also been described for many viruses like noroviruses or rotaviruses (32) and bacterial infection too. 2-FL is one of the most prominent short-chain oligosaccharides, and it is associated with the anti-infective capacity of human milk. During *Campylobacter jejuni* infection, 2-FL reduces colonization and moderates intestinal inflammation; so, the use of 2-FL as an oral agent for the prevention and treatment of this infection is being studied (33). At that place are numerous works that have assessed the clinical use of HMOs. For example, venous injection into the tail of mice of analogs of HMOs (Gal-1-4Gal-1-4Glc) demonstrated its rapid diffusion and the effect of reducing urinary infections by 10 times (34).

#### Probiotic Effect

HMOs are natural prebiotics that are resistant to digestion during their passage through the small intestine; infants lack glycolytic enzymes needed to break them down, and they reach the large intestine, where they are a selective substrate for bifidobacteria (35–37). These bacteria digest HMOs producing short-chain fatty acids (SCFAs), an energy source for the epithelial cells of the colon with a positive trophic effect on the intestinal cells. They produce an acidic milieu in the gut, making it inhospitable to potentially pathogenic microbes (37, 38); moreover, they modulate the immune system and promote the gut epithelial barrier function (22), establishing a stable ecosystem in an infant's gut. It is now recognized that a healthy microbiota has a positive impact on human health and provides benefits that go far beyond effects in the gastrointestinal tract. For instance, impairment of the gastrointestinal microbiota is associated with chronic activation of the immune system and human immunodeficiency virus (HIV) disease progression (39). In respiratory viral infections, the microbiome composition stimulates production of virus-specific CD4+ and CD8+ T lymphocytes and virus-specific antibodies (32).

The SCFA produced by the fermentation of HMOs have also well-established anti-inflammatory properties (40). Data from animal models strongly suggest a protective role for SCFAs; the SCFA receptor GPR43 was found in asthma, arthritis, and colitis models and GPR41 in allergic airway inflammation (41–45).

#### Immunomodulatory Effect

HMOs also directly modulate immune responses. Data from literature documented that SCFAs are involved in immune response regulation function; indeed, they promote the development and influence the function of regulatory T cells (Tregs), an action that limits intestinal inflammation and regulate gene expression, with consequent inhibition of interferon (IFN) production and the suppression of activation of the nuclear factor kappa Beta (NF-κB) in the epithelial cells of the human colon (46). This mechanism is responsible for the first immune inflammatory responses, and its dysregulation is found in inflammatory bowel diseases (47). HMOs may act locally on cells of the lymphoid tissues or through the systemic circulation (48, 49), where it interacts with selectins, dendritic cells (DCs), integrins, and Toll-like receptors (TLRs). Selectins are glycoproteins involved in the immune response in the initiating signal of the inflammatory cascade. HMOs induce structural modifications of selectins, reducing the immune response as adhesion, migration, leukocyte activation *in vitro*. In particular, the sialylated HMOs inhibit the adhesion, mediated by lecitins, of leukocytes to the endothelium; they limit the recruitment from the blood and suppress the infiltration of leukocytes (50); in this way, they modulate the escalation of the immune response (1).

Colostrum HMOs have been shown to modulate immune signaling pathways, including TLR3, TLR5, and IL-1-dependent pathogen-associated molecular pathways (PAMPs), and subsequently to decrease acute-phase inflammatory cytokine secretion. For example, 3'-galactosyllactose directly inhibits polyinosine–polycytidylic acid, which, in turn, decreases levels of the potent pro-inflammatory cytokine, IL-8 (27).

Studies demonstrate that the injection of milk-N-fucopentose III or milk-N-neotetraose oligosaccharide into mice caused the proliferation of a subpopulation of peritoneal macrophages that suppress CD4+ lymphocytes and the activation of natural killer (NK) cells (51) and, in the other way, the suppression of proliferation of naive CD4+ T lymphocytes through secretion of IL-10 and IL-13 by macrophages (52). Moreover, when cord blood T cells are exposed to sialylated HMOs, an increase in the number of IFN-γ-producing CD3+CD4+ and CD3+CD8+ lymphocytes have been observed, as well as IL-13-producing CD3+CD8+ lymphocytes (53). Not only *in vitro* but also *ex vivo*, data suggest that HMOs can interfere with selectins that mediate the earliest stages of leukocyte trafficking and platelet–neutrophil complex (PNC) formation (54).

### Cytokines and Chemokines

The newborn cytokine pool is lacking; instead, these mediators [IL-1b, IL-2, IL-6, IL-8, IL-10, IL-12, IL-18, IFN-γ, TNF-α, TGF-b, granulocyte colony-stimulating factor (G-CSF), macrophage colony-stimulating factor (M-CS), granulocyte-macrophage colony-stimulating factor (GM-CSF)] abound in breast milk, which is the ideal source (55, 56), and can make up for this deficiency. Accessibility of cytokines from breast milk is important to modulate the immediate and long-term inflammatory response of the infant.

Moreover, they may reach the intestine unaltered, thanks to the protease inhibitors (a1-antichymotrypsin and a1-antitrypsin) present in breast milk that preserve them from digestion. These cytokines are small proteins or peptides that act as intercellular messengers and elicit a particular response after binding to a receptor on a target cell. They represent an important biological component of maternal milk, thanks to their role in the function of immune regulation, hematopoiesis, and inflammation, and they may make up for the immaturity of the neonatal immune system and the consequent insufficient anti-inflammatory and anti-infective response. While many cytokines and chemokines have multiple functions, milk-borne cytokines may be grouped broadly into those that enhance inflammation or defend against infection and those that reduce inflammation (57).

There is large variability between cytokine concentrations among breastfeeding women, and pro-inflammatory ones are generally low. In addition, most milk T lymphocytes show the activation of CD45RO marker, in contrast to neonatal T lymphocytes, which mainly express the “naive” CD45RA phenotype. The production of cytokines in breast milk has been demonstrated by CD4+ and CD8+ T cells, which express the antigen CD45RA or CD45RO, and the percentage of cytokine-producing cells in breast milk is higher than that in cord blood (58).

High levels of IL-1 and receptors have been detected in breast milk, as well as a significant function of this cytokine has been demonstrated both *ex vivo* and *in vitro*. IL-1 receptor antagonist (IL-1RA) limits inflammation competing with the pro-inflammatory cytokine (59). The possibility of having inflammatory and anti-inflammatory systems in a balance that depends on the conditions that call for it is an extremely intriguing feature of breast milk. One of the most important mechanisms underlying the protective effect of breast milk, for example, against necrotizing enterocolitis (NEC), is the ability to attenuate the inflammatory immune response mediated by NF-κB in enterocytes. This occurs through the reduction of the activity of IL-1, which induces the innate endogenous inflammatory response, upregulating the expression of pro-inflammatory IL-8 and stimulating the pathway of the NF-κB (60, 61).

Breast milk, especially in colostrum and less in the mature one, is rich in IL-2 that intervenes in the recruitment of T cells necessary to produce an antigen-specific immune response, supporting in this function the newborn who, on the other hand, is notoriously particularly lacking in it. The high percentages of Th1-type cytokines (IL-2 and, particularly, IFN-γ) may play an important role in the differentiation of neonatal T cells in a preferential Th1, rather than Th2, pathway.

Breast milk IL-10, in maximum concentrations in the first 24 h of life, is a powerful anti-inflammatory; it is able to inhibit the activity of Th1 effector cells, NK cells, and macrophages and the production of several pro-inflammatory cytokines (IL-1, IL-6, IL-8, and TNF-α), thus attenuating the immune response (62). IL-10 increases the survival and expansion of B cells, inhibits Th1 responses, and downregulates major histocompatibility complex-II expression on monocytes, thus, limiting their antigen-presenting cell function. IL-10 has been heavily implicated in the regulation of intestinal inflammation and regulating responses to the microbiome (56).

Two effective pro-inflammatory cytokines, IFN-γ and TNF-α, are missing in the newborn. IFN-γ, secreted by activated T cells and NK cells even if at low concentrations, is involved in signaling pathways that increase intestinal epithelial barrier permeability and improves the activation of intestinal macrophages; it is also detected in higher concentrations in the ileum of patients with NEC (63, 64). TNF-α is an endogenous pyrogen; it contributes to systemic inflammation and performs a regulatory immune cell function (62).

Colostrum contains high amounts of IL-8, which is significantly reduced in the later stages of breastfeeding, an interleukin that protects the newborn from sepsis and NEC. The exposure of intestinal cells intact or with injury to the recombinant IL-8 *in vitro* has stimulated the proliferation and differentiation of the former and greater vitality in the latter, suggesting an important role in the physiological development of the intestine (65).

Human milk contains physiologically relevant concentrations (up to 1.5 mg/ml) of TGF-β, a multifunctional secretory cytokine expressed by most cell types, parenchymal cells, and infiltrating cells, such as lymphocytes, macrophages, and platelets (66). TGF-β_1_ is the predominant TGF-β isoform produced by immune cells, whereas TGF-β_2_ is most abundant in secretions including breast milk. The main activities of TGF-β are immune modulation, regulating the cellular proliferation and differentiation and decreasing pro-inflammatory cytokine expression. In particular, TGF-β immunomodulatory properties including intestinal maturation and defense, by switching immunoglobulin classes from IgM to IgA in B lymphocytes, immunoglobulin production in the mammary gland and gastrointestinal tract of the newborn, assistance with intestinal mucosal repair (67), and induction of oral tolerance (68). TGF-β also helps stabilize forkhead box P3 (FOXP3) expression and maintains the differentiation of Tregs, which inhibit immune responses and inflammation (69).

In several clinical studies, higher concentrations of TGF-β in milk are associated with the reduction of the incidence of neonatal diseases, in particular, respiratory and allergies, and a positive correlation between TGF-β levels and the neonatal production of immunoglobulins (70, 71). It has also been reported to inhibit inflammation in the intestinal epithelium and systemic production of IL-6 and IFN-γ, reducing the incidence of NEC (72, 73). In contrast, developmental defects rather than immunological dysregulation were observed in TGF-β2-deficient mice (74).

An abundant peptide in breast milk is the epidermal growth factor (EGF), which acts on the intestine by improving the barrier functions and nutrient transport and enhancing enzyme activity (75). The roles of EGF in the development of the intestine, as well as the response and repair of the intestine during intestinal injury or infection, have been reported (76). Inflammatory diseases of the small intestine are associated with an IL-18 dysregulation condition, and experimental NEC rats, treated with EGF, showed a decrease in the levels of pro-inflammatory cytokines, IL-18, on the intestinal damage site, as well as IL-18 mRNA levels. But the anti-inflammatory effect of EGF is also expressed through the upregulation of IL-10 (75, 77). EGF promotes the growth and maturation of the fetal pulmonary epithelium, stimulates the activity of ornithine decarboxylase and the synthesis of DNA in the digestive tract, and accelerates wound healing (78). EGF is highest in early milk and decreases over lactation. The average EGF level in colostrum is 2,000-fold higher and in mature milk is 100-fold higher than in maternal serum. Further, preterm milk contains higher levels of EGF than term milk (79).

Heparin-binding EGF-like growth factor (HB-EGF), a member of the EGF family of growth factors, also protects against intestinal injury in the developing intestine by binding to pathogenic bacteria (80). Rats that underwent ischemic/reperfusion injury had less pro-inflammatory cytokine expression, particularly TNF-α and IL-6, *ex vivo* (81). HB-EGF is expressed in response to hypoxia, tissue damage, and oxidative stress, including in the intestine, and has a pivotal role in tissue regeneration and repair (82).

Similarly, vascular endothelial growth factor (VEGF) is a glycoprotein present in breast milk, at higher levels in the colostrum and breast milk of mothers with preterm infants than those with term infants (83). Its primary role is mediating angiogenesis, but it has been suggested that VEGF may also have anti-inflammatory effects. In a study performed by Karatepe et al. (84), rats induced with NEC and given subcutaneous VEGF had less villous atrophy and less intestinal edema, as well as lower TNF-α and IL-6 levels when compared to NEC-induced rats that were not treated with VEGF.

CXC chemokines, powerful neutrophil activators, are present in large quantities in breast milk and perform chemotactic activities for intraepithelial lymphocytes and play an important role in the defense of the host against bacterial and viral infections (85).

### Lipids

Lipid fractions in human milk include 34–47% of saturated fatty acids (SFAs) with principally 17–25% palmitic acid, 31–43% monounsaturated fatty acids (MUFAs), 12–26% n-6 polyunsaturated fatty acids (PUFAs), 0.8–3.6% n-3 PUFA (22). Breast milk contains a high proportion of omega-3. PUFAs are involved in regulating immune and inflammatory responses by inhibiting the induction of inflammatory genes binding to the nuclear receptor, peroxisome proliferator-activated receptor (PPAR) (86). Moreover, omega-3 PUFA decreases the activity of NF-κB that induces a range of pro-inflammatory genes, including cyclooxygenase 2 (COX-2), intercellular adhesion molecule-1 (ICAM-1), vascular cell adhesion molecule-1, E-selectin, TNF-α, IL-1B, inducible nitric oxide synthase, and acute-phase proteins. PUFA in human milk can modulate immunological responses, affecting the balance between Th1 and Th2 cells and Treg and T helper cells from the acquired immune response (87). Omega-3 PUFAs change the membrane phospholipid composition by increasing arachidonic acid, subsequently decreasing the systemic inflammatory response syndrome; they also inhibit migration of leukocytes to infection sites (88). Supplementation of a combination of omega-3 and omega-6 PUFAs decreased the incidence of NEC and intestinal inflammation *via* decreased platelet-activating factor (PAF)-induced TLR4 activation in mice (89, 90). In addition, specialized pro-resolving mediators (SPMs), derived from omega-3 PUFA, specifically resolve inflammation by stopping polymorphonuclear cell migration and protect against chronic inflammatory conditions, including colitis, neuroinflammation, and arthritis (91). Omega-3 PUFAs also increase anti-inflammatory microbes, such as *Lactobacillus* and *Bifidobacterium* species (92, 93). The long-chain PUFA (LCPUFA), Docosahexaenoic Acid (DHA), and Amino Acids (AAs) are essentials as immunomodulators also by production and regulation of inflammatory cytokines, leukotrienes, prostaglandins, and thromboxanes (94).

Phospholipids contribute 1–2% of the total lipids of human milk. The major phospholipids of milk fat globule membrane are phosphatidylcholines, phosphatidylethanolamines, and sphingomyelins, and each of it contributes 20–40% of the total phospholipids (95, 96). The sphingomyelin demonstrates robust antitumor activity, may influence cholesterol metabolism, and exhibits anti-infective activity (97). Breast milk lipids have been demonstrated to inactivate a number of pathogens *in vitro*, including *Group B streptococcus* (GBS), and provide additional protection from invasive infections at the mucosal surface, especially medium-chain monoglycerides (98, 99). Free fatty acids (FFAs) and monoglycerides, the digestive products of triglycerides, have a lytic effect on several viruses and also have an antiprotozoal action, particularly against *Giardia* (99). FFAs destroy the viral lipid envelope and express an important antiviral activity against the enveloped virus, including hepatitis C virus (HCV) and HIV (100, 101).

Oxysterols are cholesterol oxidation derivatives, and they were present in breast milk in all stages of lactation; they are characterized by a broad antiviral activity, and they are involved in the innate immune response against viruses. They inhibit the viral replication, and their antiviral power has been observed against a number of enveloped viruses but also in relation to several non-enveloped viruses. They were active *in vitro* against the human rotavirus and the human rhinovirus, two relevant pediatric viral pathogens (102, 103).

### Proteins

One of the special features of breast milk proteins is that they are present in an inversely proportional way to the production capacity of the baby, and they are able to gradually decrease over time as the baby's defenses increase or increase in case of need. Breast milk contains several hundred proteins, which play functional roles in the protection of the child; they provide nutrition, stimulate the absorption of nutrients, and provide biological activities, ranging from antimicrobial effects to immunostimulatory. These molecules have broad-spectrum anti-infective power against bacteria, viruses, and fungi, as well as synergic activity with conventional antibiotics (104, 105).

The composition of human milk proteins makes them 100% assimilable by the infant's body, and they are divided into three groups: caseins, whey proteins, and those linked with the membrane of the milk fat globule. Whey protein is the main colostrum protein; while in mature milk, it is equivalent to 60%. Proteins, such as β-casein, perform important antiseptic and anti-infectious functions inhibiting the proteases of bacteria and viruses. Furthermore, the peptides produced by the digestion of α-lactalbumin show a potent antibacterial action against Gram-positive and Gram-negative bacteria. Other human milk proteins, such as SIgA, LF, and lysozyme, act as essentially anti-infectious agents for premature babies carrying out a synergistic action to inactivate, destroy, or block the attack of specific microbes on the surfaces of the mucous membranes (106). Lipoproteins can cause cell lysis in *influenza virus* infection (107).

**SIgA** provides immunological protection to the infant, while its own immune system matures (108). The concentration in colostrum is about 12 mg/ml; mature milk contains 1 mg/ml.

SIgA antibodies in breast milk are essential in the defense of the mucous membranes: prevent the entry of microorganisms in the tissues; they are anti-inflammatory and do not consume energy during the reaction (109, 110). Interestingly, 74% of bacteria in the gut lumen are coated with SIgA (111). Given this role, it is not surprising that SIgA influences the composition of the intestinal microbiome and, furthermore, promotes intestinal homeostasis by preventing inappropriate inflammatory responses to pathogenic microbes and nutritional antigens. In a recent study by Gopalakrishna et al. (112), maternal milk-fed infants had higher percentages of SIgA that were bound to bacteria compared to formula-fed infants. In addition, higher percentages of SIgA bound to bacteria in the intestine of preterm infants were associated with lower rates of NEC. Furthermore, it was observed that lower levels of SIgA-bound bacteria were inversely associated with abundance of enterobacteria among infants who developed NEC. Thus, SIgA binding to bacteria presumably plays a protective role against NEC, likely by limiting inflammation induced by *Enterobacteriaceae* (27). Specifically, SIgA molecules remain active throughout the newborn's gastrointestinal tract and impact the binding of commensal or pathogenic microorganisms, toxins, viruses, and other antigenic materials, such as lipopolysaccharide (LPS), preventing their adherence and penetration into the epithelium without triggering inflammatory responses that could be harmful to the newborn, a mechanism known as immune exclusion. High-affinity IgA and IgM antibodies transported by the polymeric immunoglobulin receptor (pIgR) may even inactivate viruses (e.g., *rotavirus* and *influenza virus*) within epithelial cells and carry these pathogens and their products back into the lumen, thereby avoiding cytolytic damage to the epithelium (113). Breast milk contains antibodies protective against *V. cholerae, Campylobacter, Shigella, Giardia lamblia*, and respiratory tract infections (114); adherence of *S. pneumoniae* and *Haemophilus influenza* to human retropharyngeal cells is blocked by SIgA antibody in breast milk (114, 115). IgM and IgG are less in human milk, but they have known immune-surveillance properties. High-avidity IgM antibodies may act as an important part in protecting the mucosal surfaces of infants against viruses and bacteria. IgG plays an anti-inflammatory role by direct binding, opsonization, and agglutination of pathogens (79, 114, 115). IgG is mainly transferred *via* the placenta from mother to fetus, but they are also produced in the mammary gland and detected in a majority of colostrum samples of mothers, adding to the much-needed immunological protection to the vulnerable infant.

### Lactoferrin

LF is the dominant whey protein in human milk (0.5 g/dl in colostrum, 0.2 g/dl at 1 month, 0.1 g/dl later) and has been demonstrated to perform a wide array of functions, protecting the newborn infant from infection and contributing to the maturation of the newborn innate and adaptive immune systems. More precisely, LF is the second most abundant protein in breast milk, and its biological functions range from antimicrobial activities against a large panel of microorganisms, including bacteria, viruses, fungi, and parasites, to regulation of cellular proliferation and differentiation, as well as anti-inflammatory, immunomodulatory, and anticancer activities (116).

LF has a high rate of survival after oral administration. In the stomach, pepsin digests and releases a potent peptide antibiotic called lactoferricin from native LF. Among all of the bioactive substances found in human milk, LF may be the one most comprehensively involved in milk's well-known anti-infective actions. A robust background of experimental research supports a leading use of LF in promoting the establishment of immune and defensive competencies in the neonate and young babies. In the last few years, solid evidence originating from a Randomized Clinical Trial (RCT) has provided clinical confirmation of these properties. LF has been demonstrated to inhibit *in vitro* replication of human *cytomegalovirus, HIV, herpesvirus, hepatitis B and C viruses, hantavirus, human papillomavirus, rotavirus, adenovirus*, and *influenza A* (117).

LF has a bacteriostatic function and cytotoxic effect against bacteria, viruses, and fungi and carries out immunomodulatory functions, helping to limit excessive immune reactions by blocking many inflammatory cytokines, such as IL-1β, IL-6, TNF-α, and IL-8 (118, 119) while stimulating the natural process and maturation of the child's immune system (85).

In detail, N-terminal receptors for LF have been identified on the surfaces of many microorganisms. The binding of LF with these receptor sites is able to induce cell death in Gram-negative bacteria through a structural alteration of the bacterial LPS, with a consequent increase in permeability and greater susceptibility to lysosomal enzymes and antibacterial agents (120).

In *E. coli* infection, LF inhibits adherence and biofilm formation potentially by binding to the lipid portions of the LPS layer and through the degradation of virulence proteins anchored to the external membrane (121). *In vitro* LF is able to prevent the formation of the biofilm of *Pseudomonas aeruginosa*. In fact, in the absence of iron, this bacterium seems to have poor mobility, which results in the impossibility of surface adhesion (122). The proteolytic activity of LF is able to inhibit the bacterial growth of *Shigella flexneri* or enteropathogenic bacteria (5).

It has also been reevaluated for its antiviral capabilities, and some of them are expressed through the attachment of LF to surface proteoglycans, such as heparan sulfate, through its N-terminus glycosaminoglycan-binding domains, thus blocking the entry of certain viruses into the cell, guaranteeing an arrest of the infection in its early stages (5, 123). In a study conducted *in vitro* on the activity of LF against the main pathogenic viruses (124), the results demonstrated LF inhibitory activity against a large variety of viruses. LF prevents DC-mediated HIV type 1 transmission by blocking the DC-SIGN-gp120 interaction (125) and human HCV infection, and a direct interaction between LF and E1 and E2 HCV envelope proteins has been proven (117).

LF can be considered a regulator of both innate and acquired immunity (126). The effects of LF on the innate immune response are related to its ability to bind to a variety of targets, first of all PAMP, present on pathogens (e.g., LPS on Gram-negative bacteria and peptidoglycans on Gram-positive bacteria), and thereby it competes with LBP, inhibiting the release of pro-inflammatory cytokines (118, 122, 127). Data indicate that the interference of LF in the LPS–CD14 interaction may inhibit the expression of E-selectin, ICAM-1, and IL-8 by human umbilical vein endothelial cells, showing that the LF may also modulate the recruitment of immune cells by interfering with the expression of endothelial cell adhesion molecule, required for the recruitment of these cells to sites of inflammation (118, 127). LF is also capable of enhancing the activation of immune cells. Following bacterial invasion, LPS binds to TLR4 on sentinel cells to cause the release of potent cytokines, including TNF-α, IL-1, and IL-6 (127, 128). These molecules will activate and modify the permeability of endothelial cells to allow the passage of complements and antibodies and recruit neutrophils to the site of inflammation. Activated neutrophils will release LF from their secondary granules to exert its direct microbicidal effects (127). LF may also enhance the cytotoxic functions of NK and lymphokine-activated killer cells, potentially through binding to RNA and DNA. On macrophages, there are LF receptors, and the LF binding activates macrophages to release pro-inflammatory molecules, e.g., TNF-α, IL-8, and nitric oxide (129), and increases their phagocytic activity when infected (130). For example, it increases phagocytic killing of *Staphylococcus aureus* (131).

LF plays an important immunomodulatory role in activation and antigen presentation by antigen-presenting cells (APCs), modulation of T cell development (132, 133), and maturation of DCs, enhancing IL-8 and CXCL10 release, decreasing antigen internalization, and increasing their capacity to trigger proliferation and release IFN-α (134).

When administered orally, LF has the ability to restore the host T cell compartment by an increase in splenic cellularity and enrichment of CD3+ CD4+ T lymphocytes, suggesting a possible function for LF in the reconstitution of the cellular immune response (135).

During the last years, literature data confirmed the crucial role of LF in the development of the intestinal microbiome, an important finding for hospitalized term and preterm infants at risk of invasive infection and NEC (136). It was shown that the integration of 100 mg/day of LF in preterm births reduces the onset of delayed sepsis and intestinal necrosis (127, 137, 138). LF also acts as a natural and specific inhibitor of the Urokinase Plasminogen Activator (uPA)-mediated plasminogen activation (139); high levels of LF were reported to be secreted by apoptotic cells (140). In apoptosis, LF might be necessary to downregulate the extracellular plasmin activity to avoid unwanted degradation of the surrounding tissue, inappropriate cell migration, or activation of proenzymes; the serum levels of LF might further increase during pregnancy. Thus, an anti-fibrinolytic property of LF may provide an intriguing clue to the reported higher risk of thromboembolism during pregnancy (141, 142). This view is especially relevant if we consider that thromboembolism is one of the most destructive mechanisms currently recognized in COVID-19 (143, 144). LF has been shown to act as a natural barrier of both respiratory and intestinal mucosa or reverting the iron disorders related to the viral colonization as an antiviral, anti-inflammatory, and immunomediator (145).

### Lactadherin

Lactadherin has antimicrobial and antiviral effects and plays an important role in the immune defense. Lactadherin can be considered a multifunctional glycoprotein involved in the regulation of many biological and physiological processes, such as angiogenesis, atherosclerosis, hemostasis, phagocytosis, and tissue remodeling. The intrinsic endogenous activities of lactadherin include promotion of mucosal wound healing and attenuation of intestinal inflammation *ex vivo* and *in vitro* (146–148). Lactadherin also induces IL-10 and TGF-β release from Tregs and promotes intestinal DC development (146). It enhances the ability of macrophages to phagocytize apoptotic cells, thereby ameliorating inflammatory processes induced by NF-κB and mitogen-activated protein kinase (149). It activates the signal transducer and activator of transcription 3–suppressor of cytokine signaling three pathways, directly quenching LPS induced TNF-α production (146, 147, 149). Indeed, it has been reported that recombinant lactadherin may attenuate sepsis-induced apoptosis (150).

Milk lactadherin is present in the intestines of breastfed infant and could gain access to the circulation with its strong anticoagulant effects. It can mediate clearance of phosphatidylserine-expressing procoagulant platelet-derived microvesicles (151). Given the homology of its C2 domain to coagulation factors VIII and V and the competition between them for platelet membrane-binding sites, lactadherin could function as an anticoagulant and thereby regulate hemostasis. Many diseases induce strong procoagulation processes, including sepsis, suggesting other possible protective activities (152). Thus, orally ingested lactadherin could potentially be used for the prevention and treatment of intestinal injury in infants.

### Human β-Defensin

Human milk contains small bioactive peptides, including defensins; of these, human β-defensin (HBD)-1 may act synergistically with other peptides present in breast milk. Also, HBD-1 acts as a chemotactic agent to recruit DCs, T cells to mucosal surfaces (respiratory, gastrointestinal, and nasopharynx), thus acting as a link between innate and adaptive immunity for the neonate (153). HBD-2 is present at 8.5 mg/ml in colostrum and 1 mg/ml in mature milk, and it has antimicrobial activity against pathogenic bacteria (154). This suggests that the presence of HBD-2 in milk may help to defend both the mammary gland and the infant intestine. HBD-2 enhances TLR4/CD44 against intestinal pathogens (1). Low concentrations of HBD-2 are associated with lower TLR4-lymphocyte antigen concentrations and more severe NEC (155). HBD-2 also suppresses TLR7 expression (54) that is stimulated by single-stranded RNA, and during a virus infection, it mediates signaling that leads to the release of IFN (156). HBD-2 in milk may contribute to decrease, in the long-term, the risk of gastrointestinal inflammatory diseases in the breastfed infant and to reduce the risk of breast cancer in mothers who had breastfed (157). Low levels of HBD-1 and HBD-2 in preterm infants are associated with increased incidence of intestinal pathology and onset of NEC. Animal models have shown that depletion of Paneth cells rich in defensins followed by enteric infection in test animals resulted in a clinical picture akin to human NEC (158). A synthetic analog of HBD (brilacidin) is tested in a phase II trial for the treatment of acute bacterial skin infections and in preclinical phase for otitis and ocular infections (159).

### Osteopontin

It is a multifunctional glycosylated and heavily phosphorylated acidic protein. The average concentration in breast milk is comparatively high (~140 mg/L) and about half maximal levels are maintained beyond 1 year of lactation (160). It also plays an important role in immune activation and immune regulation by acting as chemotactic agent and stimulates both pro- and anti-inflammatory processes. It enhances B lymphocyte immunoglobulin production and proliferation and also influences cell-mediated immunity by inducing Th1 cells (161). Furthermore, it also has been shown to form complexes with LF and act as a carrier for other immunomodulator proteins to enhance their competencies (162). Infants fed formula supplemented with osteopontin (OPN) had significantly low serum concentrations of the pro-inflammatory cytokines; the cytokine profile is more similar to that of breastfed infants and days of illness were significantly lower, and these data strongly suggest that OPN affects immune function in infants, conferring health benefits (163).

### Antiprotease and Antioxidant Factors

Indeed, oxidative stress may be involved in serious diseases in premature infants, including NEC, chronic lung disease, and retinopathy of prematurity (ROP) (164). Human milk contains anti-proteases, including α-1-antitrypsin, α-1-antichymotrypsin, and elastase inhibitor, which limit the ability for pathogens to enter the body, thereby limiting inflammation locally (109). It also contains various antioxidative agents, including enzymes [catalase, glutathione peroxidase (GPx), and superoxide dismutase (SOD)], constituents of antioxidative enzymes (e.g., Cu and Zn), vitamins (A, C, and E), and binding proteins (e.g., LF) (165).

*In vitro* studies suggest that human LF decreases the intensity of free radical processes (166), and adiponectin (4.2–87.9 ng/ml) enhances the antioxidative potential of cells to counteract the increase in intracellular reactive oxygen species (ROS) during the hypoxia–reoxygenation-induced apoptosis (167). Animal studies showed that lysozyme participates in the suppression of ROS and strengthens resistance to chronic exogenous oxidant stress (168).

Melatonin is interesting in the context of milk bioactive compounds due to its pleiotropic actions. Its protective effects have been demonstrated against cellular aging due to its antioxidant effects stimulating the expression of SOD, catalase, and GPx (169). Tryptophan found in breast milk and its metabolites would seem to possess strong antioxidant properties and good anti-product abilities (170). Lozano et al. (171) in 2014 found the antioxidant capabilities of other valuable molecules naturally found in human milk, such as coenzyme Q10, tocopherol, fatty acids, and IL-10. Breast milk contains adequate amounts of selenium, a necessary microelement for important antioxidant enzymes, defined precisely as Se-dependent molecules, including GPx and glutathione reductase.

### Cells

**Figure 2:**
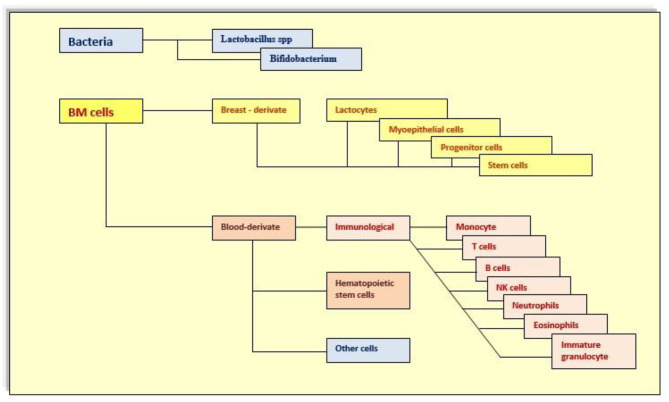
Cells in human milk.

The heterogeneous mixture of breast milk cells (Figures 1, 2) includes leukocytes, epithelial cells, stem cells, and bacteria. Certainly, cells of human milk are not insignificant components, but their function is still unclear. Leukocytes are the most widely studied cell types due to their protective properties and their ability to infiltrate the infant's tissue. About 80% of the cells in early milk are breast milk macrophages, and phagocytosis of human milk components transforms these monocytes into potent breast milk macrophages with unique functional features, including the ability to differentiate into DCs that stimulate infant T-cell activity (4, 169, 171–173). This capability provides broadly powerful protection against pathogens while stimulating the development of the infant's own immune system.

Human breast milk is rich in microRNAs (miRNA), and so far, more than 386 different miRNAs were identified (174). Compared with other body fluids, milk is one of the richest sources of miRNA, which are present in all three fractions of human milk, including cells, lipids, and skim milk (175). miRNA plays a key role in regulating the immune system, including T and B cell development (176, 177), release of inflammatory mediators (178), proliferation of monocytes and neutrophils (176), and differentiation of macrophages and DC. mRNA might be engaged in the epigenetic regulation of stem cells fate and function (178, 179).

Speaking of stem cells, they are being studied, but their roles are presently little known. It is considered that they can play a cardinal part in the repair of tissue or organ damage caused by several types of insults (180). This is also a really important factor to recto, considering that the deleterious effects of COVID-19 infection are precisely related to epithelial damage and the consequent repair mechanisms are sometimes seriously distorted (181, 182).

## Breast Milk and Viral Infection

**Figure 3:**
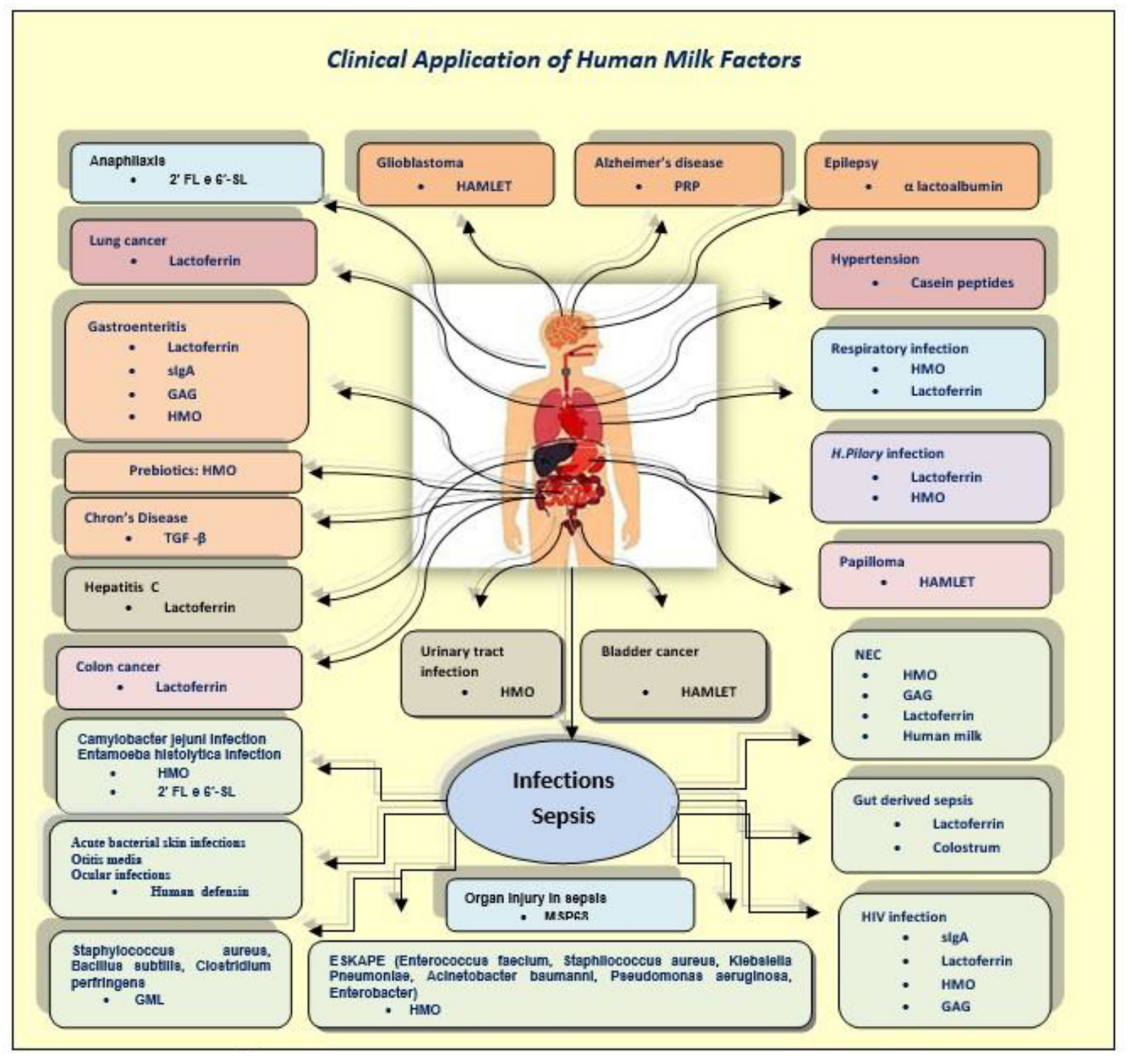
Da RJH 2014 modified.

After considering all the active components of breast milk, the second part of this study wants to underline their major antiviral effects (Figure 3).

### Breastmilk and Respiratory Syncytial Virus

Literature data confirm nowadays that viral respiratory infections are the major cause of children hospitalization worldwide, and a lot of studies were conducted to demonstrate that breastfeeding has an important preventive role, supporting the postulate that the risk of infant morbidity for viral acute respiratory diseases is negatively associated with the duration of breastfeeding (183). Some authors suggest that human milk reduces the risk of respiratory infection in newborns and the oxygen medication during bronchiolitis because the decrease of chemokine concentration in the airway and the increase of anti-inflammatory mediators like IFN-γ probably mean stronger immune response in breastfed infants (184, 185). A crucial role in human milk was played by LF that may decrease the production of chemotactic cytokines and neutrophil chemotaxis (186). Also, oligosaccharides and procathepsin D can influence the neutrophil activity modifying the neutrophil adhesion, infiltration, and activation (187). The relationship between mother and baby during breastfeeding is bidirectional; indeed, the mother exposure to the baby's infection increases the production and the passage of anti-inflammatory constituents through the milk (7, 188, 189).

### Breast Milk and *HIV* Infection

A lot of studies demonstrate that innate and adaptive immune mediators of human milk inhibit the *HIV* infection, preventing the mother-to-child transmission and inhibiting the virus activity; an important role is played by HMOs, LF, and other innate factors (5). Specifically, HMOs block the binding of *HIV* on human DCs; they also directly modulate the immune cell responses (1). LF inhibits the virus interaction with CD4 receptor, reducing inflammation (190). Soluble TLRs (sTLRs) interact with specific *HIV* structural proteins, inhibiting the activation of the virus (191). Breastfeeding nowadays represents one of the most successful prevention interventions for *HIV* (5).

### Breastfeeding and Gastrointestinal Infections

*Noroviruses* are the most frequent causes of acute gastroenteritis among children, and human milk with its HMOs is able to protect breastfed infants against this infection. Recent studies demonstrate that the *Norovirus* interacts with histo-blood group antigens (HBGAs), and this binding is important for virus attachment to cells causing infection (192, 193). HBGAs have been found in saliva and on epithelial cells, and some authors demonstrate that HMOs mimic their structure (194). Weichert et al. (193) in their study showed how HMOs, specifically fucose, can block the HBGA binding site, inhibiting the virus interaction. To account that human milk contains 20 to 30 mg/l of fucose, the authors declare that breastfeeding works as a *norovirus* antiviral (183, 193). It is also well-known that *rotavirus* infection represents the most dangerous infection during childhood, specially between 6 months and 2 years; it can cause a very large number of gastroenteritis episodes and hospitalization, and it can be estimated that the virus can cause approximately 500,000 deaths in children under 5 years (4, 195). The infection is due to an interaction between the virus and an enterocyte, resulting in alteration of absorption and secretion of the intestinal tract (196). The World Health Organization and the major scientific society recommended that infants should be exclusively breastfed for the first 6 months (197), and in a recent review, the authors showed an interesting positive role of breastfeeding in reducing *rotavirus* infection prevalence among exclusively breastfed children (196). HMOs again have a crucial role. A recent clinical study has demonstrated that the *rotavirus* infection mechanism is similar to that of the *norovirus* one; indeed, an *in vitro* study documented that the *rotavirus* interacts with some type of HBGAs (32). HMOs at the same time block the binding of *rotavirus* to epithelial cells and modulate the apoptosis and the differentiation of epithelial cells, modulating intestinal susceptibility (198). HMOs, in *norovirus* infection, might work as specific antivirals, blocking the HBGA binding site and at the same time blocking the binding of *rotavirus* to epithelial cells (8, 187, 198, 199). According to some authors, HMOs play together with newborn microbiota, resulting in a better infection protection (200, 201). Therefore, the bioactive human milk mediators can improve the immune response after vaccine (198). During the last 20 years, scientists conducted a lot of studies about other human milk constituents and their impact on *rotavirus* infection. Before HMOs, the attention was for mucin proteins, butyrophilin, lactadherin, and LF. A lot of studies have shown the antirotavirus activity of these components, reducing virus infectivity and, in some cases, the interaction between the cells and the virus (202). Human milk oxysterols have broad antiviral activity inhibiting viral replication and are now considered involved in the innate immune response against viruses; *in vitro* studies have demonstrated a powerful activity against *rotavirus* and *rinovirus* and are considered an emerging class of antiviral effectors (102, 103).

Breast milk, in conclusion, represents a strong protection against a lot of enteropathogens because it is rich in immune and non-immune mediators; a large number of studies showed an important positive effect in diarrhea morbidity and mortality due to the mixture of all immunologic components (203).

### Breastfeeding and *Influenza Virus*

*Influenza viruses* are three types of pathogens, A, B, and C; type A is the most frequent cause of influenza in humans, in children too. It can cause respiratory symptoms so the upper and the lower respiratory tracts are the specific infection sites. Generally, after the virus interaction with the respiratory tract, serum immunoglobulins are produced. It is known that these immunoglobulins protected the newborn through breast milk, but an important role is played by other immune mediators like antiviral cytokines that modify the respiratory epithelial cells blocking the interaction with virus (204). Prostaglandins PGE2 and PGEα inhibit the growth of *parainfluenza virus 3* (205). LF can inhibit virus absorption and cell penetration too (206). Breast milk lipoproteins can cause cell lysis. Macrophage, IL, and IFN-1 modulate the immune response against viruses; in fact, a study demonstrates that high levels of IFN in human milk can mitigate influenza virus infections, especially H1N1 (204). Because of its structure, influenza virus interacts with HMOs, modifying virus affinity for epithelial cells (207). The importance of breast milk for infection prevention is due mostly to its biodynamic constituents.

In the last years worldwide, there were big viral infection outbreaks like avian influenza, Ebola virus, Zika virus, MERS *coronavirus*, and nowadays the big pandemic COVID-19. Mothers infected were always encouraged to breastfeed.

According to literature data, breast milk can be a source of viral infection, for example, Zika infection and others, but the bioactive mediators carried by milk can directly modulate the real infectivity of viruses (208, 209). The potential risk of infection should be compared with the large benefits of breastfeeding during the first 6 months of life.

### *SARS-CoV-2* and Breastfeeding

Generally, 229E, OC43, and HKU1 *coronavirus* are the common causes of cold. Instead, Middle East respiratory syndrome coronavirus (MERS-CoV), SARS-CoV, and the new SARS-CoV-2 cause severe respiratory symptoms with high pathogenicity and mortality in humans; SARS-CoV-2 generated a new pandemic disease called COVID-19 or coronavirus disease 2019 (210). This infection was due to a new type of *coronavirus*; these viruses have rapidly evolved and changed during the past years. SARS-CoV-2 is an enveloped, single-strand RNA virus that infected humans and also animals. They are able to alter the tissue specificity and to mutate rapidly in different epidemiological situations (211, 212); this genome is similar to the other *coronaviruses* and encodes the structural protein involved in viral replication, as spike (S), envelope (E), nucleocapsid (N), and membrane (M) as well as non-structural and accessory proteins. This will result in a large number of suitable therapeutic targets (213). Literature data suggest that the spike protein mediates attachment of the virus to the cell surface by interaction with its glycoprotein receptor (211, 212). A recent study found that a metallopeptidase, the angiotensin-converting enzyme 2 (ACE-2), could represent a receptor for the virus mediating the entry into host cells; to facilitate this entry, a large number of coreceptors are required (213). Moreover, taking into account the similarities between CoV and CoV-2, the crucial role of the heparan sulfate proteoglycans (HSPGs) on the cell might be thought like a mediator for virus binding and entering the host cell.

Nowadays, the new SARS-CoV-2 can cause a severe respiratory disease with fever, dry cough, severe dyspnea, and in some cases diarrhea; this clinical manifestation can evolve into pneumonia, metabolic acidosis, septic shock, bleeding, and unfortunately death (214, 215). In particular, SARS-CoV-2 has a high rate of contagiousness, and it can be transmitted through direct contact, aerosol droplets, and fecal–oral way, and both symptomatic patients and asymptomatic ones can spread viruses (216). Little is known about maternal–fetal transmission; during SARS–CoV, studies confirmed the possibility of vertical transmission, and pregnancy has been complicated by spontaneous abortion, oligohydramnios, and intrauterine growth restriction (IUGR) probably due by fetal thrombotic placenta vasculopathy with fetal vascular malperfusion (217). Stumpfe et al. (218) in their review suggest that CoV infection after the first trimester can reduce the placenta function. Limited information about MERS infection suggests better outcome; only 13 cases were reported (219). Recent studies documented that there is no evidence of transplacental transmission (144, 220–225), but more data are needed and some authors reported COVID-19 positive test 48–72 h after birth (226, 227). For real, it is unclear how CoV-2 interacts with the fetus during labor and delivery (228).

Important for the transmission are perinatal and postnatal routes through aerosol and droplets. For now, any evidence of the presence of CoV-2 in breast milk, but antibodies against the virus have been found. So, United Nations Children's Fund (UNICEF), WHO, Centers for Disease Control and Prevention (CDC), and other societies suggest to continue breastfeeding, but it is important to take all the respiratory precautions to prevent the spread of the virus (228). CDC underlines that the virus has not been found in human milk, and for now, it is unknown if affected mothers can transmit COVID-19 through their milk, so breastfeeding and the provision of human milk are strongly recommended by scientific organizations. To account for the benefits of breast milk, and the lack of evidence about the presence of the virus in the milk, it is important to continue breastfeeding with the necessary precaution like facial mask, washing hands, and disinfecting contaminated surfaces. Some authors suggest personalized prenatal intervention to explain to family the important role of human milk with its antibodies, HMOs, LF, and other mediators for the prevention of viral infection (229).

The American Academy of Pediatrics (AAP) (230) affirmed that breastfeeding can help to protect newborns against viral infection not only by the active mediators of human milk but also by avoiding the potential spread of virus through the handling of formula, bottles, and other supplies (231).

It has been reported that the immune response is essential to inhibit viral infection; this mechanism is mediated by genes that encode active mediators, like LF (232). During SARS-CoV, Reghunathan et al. (233) demonstrated increased levels of LF that through NK cell activity stimulated the immune response. LF blocks the interaction between CoV and HSPGs, inhibiting the binding with host cells (234). Moreover, it blocks virus spike protein binding with ACE-2, inhibiting the attachment and the fusion between virus and host cells (235). In the same way, LF could interfere with CoV-2 HSPGs and ACE-2 pathways. The study of Lang et al. (232) suggests that LF can inhibit the entry of SARS pseudovirus into host cells. It is known that the virus adhesion to the host cell is necessary for infection, and CoV-2 utilizes glycoprotein and ACE-2 metallopeptidase receptors; in the presence of receptor analogs, there will be a competition between these mediators and the virus. HMOs could interfere with virus binding, reducing the change of developing disease. No data are available, but probably future research could confirm this theory as in the case of influenza viruses. Pandey et al. (207) demonstrated that HMOs have an antiviral activity against a large number of avian influenza viruses, blocking the pathogen attachment to the host cells maybe in the same way this human milk mediators could inhibit the CoV-2 adhesion with host cells.

## Breast Milk Factors: A New Topic of COVID Research

**Figure 4:**
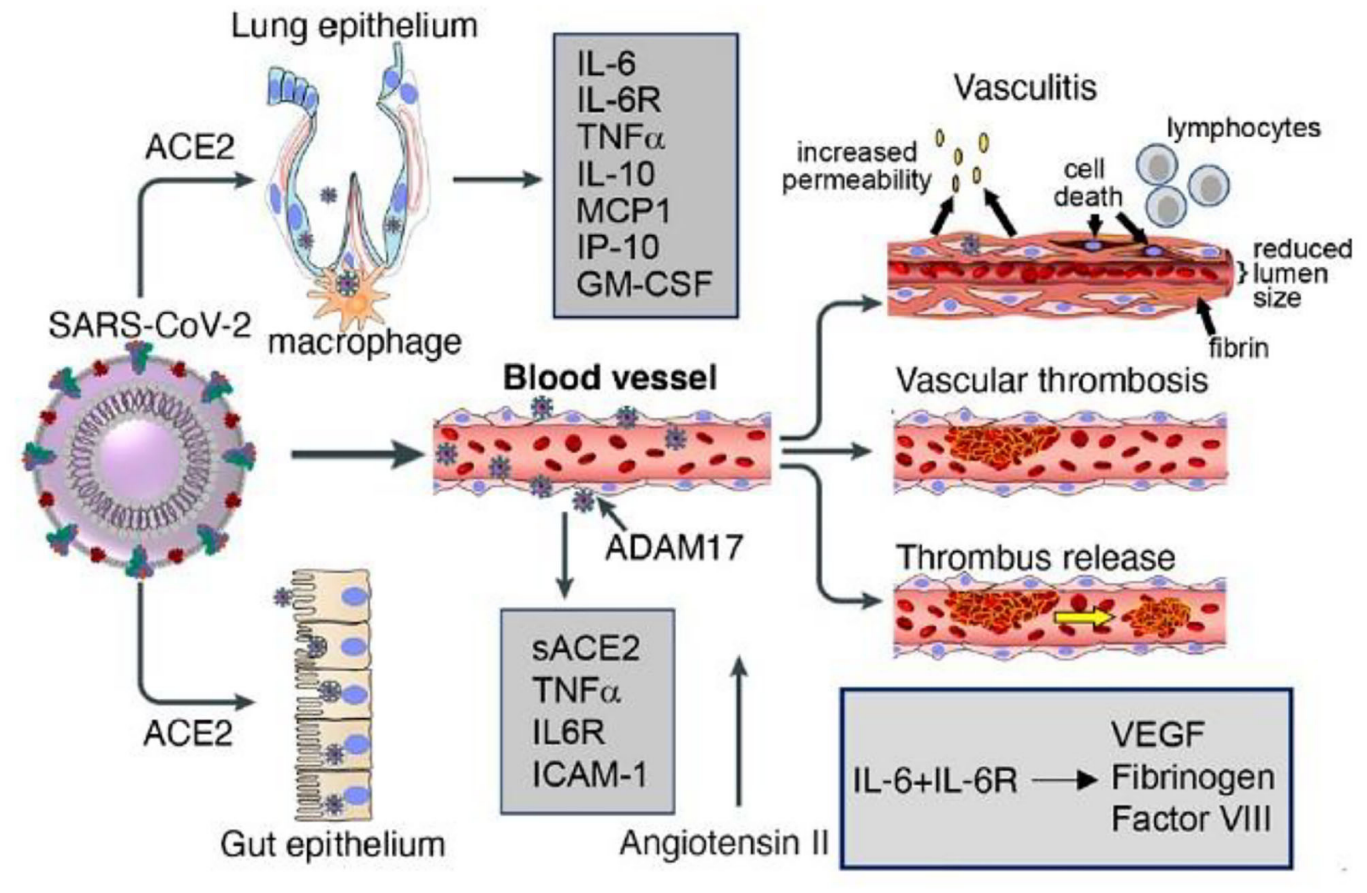
Lung and gut epithelia, macrophages, and vascular endothelia are infected by SARS-COV-2 in COVID 19. Soluble (s) ACE2, inflammatory cytokines, cytokines receptors, chemokines and other factors are released by virus binding and cell infection. Vascular pathology in COVID 19 includes vasculitis, associated with endothelial cell death, increate vascular permeability, recruitment of inflammatory lymphocytic cells, fibrin deposition and reduction of lumen size; vascular thrombosis; and vascular embolization. Labò et al. (236).

In this section, we reported the evidence *in vivo* and *in vitro* that shows how the bioactive components of breast milk are capable of performing functions theoretically useful in all pathogenic events recognized and described in the COVID disease (Table 1, Figure 4). Based on mechanisms regarding the effects of the milk factors, specific treatments could be developed in order to regulate the action of the immune system stimulating the specific contrast of the virus and blocking the start of a disproportionate and harmful immune response, in this disease, which continually shows new clinical aspects.

### Virus Invasion, Replication, and Release

CoV-2 gets inside the respiratory system through droplets (237). The viral glycoprotein on its capsid, the “spike protein,” binds the ACE-2 receptor and the RNA genome enters the host cell, mostly the alveolar cells, *via* receptor-mediated endocytosis. ACE-2 is a transmembrane protein expressed by lung alveolar epithelial cells, but also enterocytes and vascular endothelial cells (236). The spike protein is composed of two (S1 and S2) subunits. Once S1 binds the receptor-binding domain (RBD) of the ACE-2 receptor on the target cell, the HR1 and HR2 domains of the second subunit bring the cell and virus membranes in proximity to each other in order to facilitate their fusion and consequently the infection. Two spikes have been shown to bind an ACE-2 dimer at the same time. Simultaneously with the binding of the spikes to the ACE-2 receptor, the serine proteases type II transmembrane serine protease (TMPRSS) and furin carry out a modification of S2 structure, which allows the virus penetration into the host cell. Both endothelial cells and pneumocyte II express high concentrations of ACE-2 and TMPRSS. TMPRSS2 and furin are both essential for proteolytic activation and spread of SARS-CoV-2 in human airway epithelial cells (238, 239).

LF blocks the interaction between CoV and HSPGs, inhibiting the binding with host cells; moreover, it blocks virus spike protein binding with ACE-2 inhibiting the attachment and the fusion between virus and host cells. Therefore, LF has been listed as a drug candidate for the treatment of SARS disease (232, 234). Based on this concept, an ongoing clinical trial is studying the effect of a dual combination treatment with local solubilized intranasal spray formulation and oral administration (145).

Milk produced by infected mothers is a source of anti-SARS-CoV-2 IgA and IgG and neutralizes SARS-CoV-2 activity (240, 241). Mothers have unique profiles in their milk sIgA antigen-binding domains, the part of the antibody that recognizes and binds to the SARS-CoV-2 virus. It would be useful to determine whether this variability relates to which part of the virus the antibody recognizes, to the neutralization ability of the antibody, or to the severity of the illness in the mother (242).

However, other researchers have verified that the main antimicrobial components of breast milk, such as LF and IgA antibody, showed limited anti-*coronavirus* activity, but whey protein is a direct-acting inhibitor of SARS-CoV-2 infection by blocking viral attachment, entry, and even post-entry viral replication. This suggests that other factors of breast milk may play the important anti-COVID-19 role (243). Even with the limits of a preliminary and preprint report, and with the necessity of further investigation of its molecular mechanism of action in the context of SARS-CoV-2 infection, the data are interesting.

### Hyperimmunity and Cytokine Storm

The interaction between the viral antigens and the host immune cells (244) results in activation of pro-inflammatory responses as vasodilation, an increase of vascular permeability and accumulation of humoral factors.

#### Toll-Like Receptor Pathway

The elevated inflammatory response against CoV-2 is represented by an abundant expression of pro-inflammatory cytokines, the products of the TLR signaling pathway (245, 246). TLR4 possesses a strong binding affinity to spike protein followed by TLR6 and TLR1.

Therefore, the interaction between TLR4, the most efficient innate immune receptor that induces proinflammatory responses after binding with the pathogenic ligand (247), and spike protein could be one of the reasons behind the immunopathological manifestation of COVID-19 (248, 249). The interaction could be more complex, involving TLR3, TLR5, TLR6, and TLR7 (245); specifically, TLR7 agonist has been proposed as a therapeutic option (250).

In a study with TRAM-TLR3/TLR4-deficient mice, it was demonstrated that affected mice are more susceptible to SARS-CoV than wild type; mice deficient in the TLR3/TLR4 showed increased weight loss, increased mortality rate, reduced lung function, increased lung pathology, and higher viral titers (251). Some authors have suggested a line of research that aims to identify signaling pathways altered during SARS-CoV-2 infections, and this may help to unravel the most relevant molecular cascades implicated in biological processes mediating viral infections and to unveil key molecular players that may be targeted (252).

Aberrant pro-inflammatory cytokine, chemokine, and IFN-stimulated gene signaling pathway were noted following infection in modified mice that were similar in human patients with poor disease outcomes following a SARS-CoV or MERS-CoV infection (251). These findings highlight the importance of TLR adaptor signaling in generating a balanced protective innate immune and targeting aberrant monocyte/macrophage activation to reduce the severity of the *coronavirus*-induced symptomatology.

There are two groups of TLR modulatory human milk components. One group includes lacto-*N*-fucopentaose III (LNFP III) and sialyl (α2, 3) lactose (3-SL), which increase TLR signaling. Another group includes sTLRs, soluble cluster of differentiation (sCD), lactadherin, and LF, β-defensin 2, and several oligosaccharides, which depress TLR signaling pathways (31).

#### Pro-Inflammatory Feedback Loop

CoV-2 infects the epithelial and endothelial cells and alveolar macrophages, triggering the generation of pro-inflammatory cytokines and chemokines (236, 253), including IL-6, IL-10, IL-1, IL-8, TNF-α, macrophage inflammatory protein (MIP1α, MIP1β, and MIP1), that recruit immune cells, neutrophils, and macrophages to the site of infection. As in other lung infections, severe lung inflammation is perpetrated by this invasion of neutrophils and macrophages into the alveolar space, which promotes further inflammation and establishes a pro-inflammatory feedback loop that results in damage to the endothelial and epithelial lung layers (254, 255). Similarly, during *influenza virus* and *RSV* infections, increased numbers of neutrophils in the lungs contributed to worse outcomes (256–258).

Human milk contains a well-developed anti-inflammatory system that includes several bioactive factors.

The HMOs of human milk inhibit the adhesion, mediated by lecithins, of leukocytes to the endothelium, a critical step in the expansion of the inflammation (1, 50), and suppress the infiltration of leukocytes and the immune response.

IL-10 of breast milk is a powerful anti-inflammatory, since it is able to inhibit the activity of Th1 effector cells, NK cells, and macrophages (62) and the production of several pro-inflammatory cytokines (IL-1, IL-6, IL-8, and TNF-α), thus attenuating the immune response. Human breast milk dramatically suppresses IL-8, the pro-inflammatory cytokine that is significantly in the severe form of the CoV-2 disease (236, 253).

Milk fat globule-EGF-factor VIII (MFG-E8) inhibits excessive neutrophil infiltration of organs, leading to moderate attenuation of organ injury and an improved survival rate in septic mice (259).

The inflammatory environment in COVID-19 enhances the production of reactive oxygen species (ROS), impairs lung barrier function, and increases vascular permeability, and, where ARDS is prolonged or unresolved, it can lead to fibrosis (260). Antioxidants are a significant part of the anti-inflammatory system in human milk and can counteract just the excessive production of ROS due to hyperoxia, reperfusion and/or inflammation, and ROS-induced tissue damage (166).

#### Cytokine Storm

The most serious aspects of the CoV-2 disease are related to an excessive immune reaction in the host called sometimes “cytokine storm” (143, 236, 245, 260–263). The effect is an extensive tissue damage. The protagonist of this storm is IL-6. It is produced by activated leukocytes and acts on a large number of cells and tissues. It is able to promote the differentiation of B-lymphocytes and the growth of some categories of cells, inhibit the growth of others, and stimulate the production of acute-phase proteins. In the lungs of human patients with fatal SARS, elevated levels of IL-6 were detected (264). In CoV-2 disease, increased levels of IL-6 were significantly associated with high hs-TnI levels (265), a cardiac-selective biomarker of myocardial infarction and injury (266).

However, anti-inflammatory cytokines such as IL-4 and IL-10 are also increased in COVID-19 patients, and their levels are also related to disease severity (143, 267). This demonstrates the close relation between pro- and anti-inflammatory pathways. Breast milk factors have known and unused implications in the complex system of inflammation.

Experimental studies proved that adiponectin expression is negatively regulated by pro-inflammatory cytokines, including IL-6 and TNF-α, whereas adiponectin modulates the action and production of TNF-α in various tissues, while hypoadiponectinemia is associated with increased IL-6 levels (268, 269).

Intraperitoneal injection of human milk hyaluronan suppresses immune activation in a murine model of colitis (270) and promotes the proliferation of anti-inflammatory IL-10-secreting Tregs (271).

Supplementation of the formula with TGF-β2 downregulated the pro-inflammatory cytokines as well as the number of activated lymphocytes, eosinophils, mast cells, CD80, and CD86 DCs. TGF-b2 suppresses macrophage inflammatory responses in the developing intestine and protects against inflammatory mucosal injury. Enterally-administered TGF-β2 protected mice from experimental NEC-like injury (272). Feeding formula deficient in TGF-β2 resulted in accumulated IL-18 protein release from intestinal epithelial cells and IL-18 mRNA upregulation (273, 274).

Enterocyte IL-18 is upregulated in Th1-associated diseases such as Crohn's disease (275).

The remission rates following the use of TGF-β-based preparations for the treatment of Chron's disease in adult patients are higher than 80%, and when correlated to traditional treatments with cortisone, they were equally effective but with fewer side effects and a better nutritional profile (277).

It was demonstrated that posttreatment with MFG-E8-derived peptide (MSP68) significantly reduces the inflammatory response, decreasing IL-6 levels, and attenuates organ damage in septic mice (259). It was also shown that MSP68 treatment improves the histopathology and the integrity of the lung tissue and decreases lung apoptosis. MFG-E8 has been referred to as a potent therapeutic agent in sepsis-associated acute kidney injury by improving renal functions. The treatment with recombinant mouse MFG-E8 attenuated the renal function biomarkers, which was accompanied by the reduction of renal tissue histological damage and inhibition of the expression of pro-inflammatory cytokines, chemokines, and cell adhesion molecules in the renal tissues (259).

### Hypercoagulation

Another pathogenic event of the COVID-19 is procoagulant effect and diffuse thrombosis with particular involvement of the pulmonary but also cardiac vessels (236). Evidence supports the strong prognostic importance of the coagulopathy impact on affected patients (182, 216, 278, 279).

An additional LF property that was identified in an interesting study is to act as a natural and specific inhibitor of the uPA-mediated plasminogen activation. High levels of LF were reported to be secreted by apoptotic cells (140). In apoptosis, LF might be necessary to downregulate the extracellular plasmin activity to avoid unwanted degradation of the surrounding tissue, inappropriate cell migration, or activation of proenzymes.

This topic is relevant if we consider that pulmonary embolism, intestinal ischemia, and vasculitis are serious manifestations of COVID-19 (280–282) (Figure 4).

A hypothetical possible role could be played also by lactadherin that could function as an anticoagulant and thereby regulate hemostasis, thanks to the homology of the C2 domain to coagulation factors VIII and V, and the competition between them for platelet membrane-binding sites (152).

### Multi-Organ Damage

The endothelium may appear to be the battlefield for COVID-19 (283). Systemic inflammatory vasculitis, secondary to endotheliosis, emerges to be the most feared complication in a SARS-CoV-2 infection, and the endothelium could be a secondary target of SARS-CoV-2 infection. The ubiquitous distribution of ACE-2 in blood vessels may underlie the multi-organ pathology of COVID-19 (265). The endothelial dysfunction results in reduced vasodilation, pro-inflammatory state, and prothrombin properties (159).

Several factors of human milk have shown protective and reparative abilities of the endothelium (1, 18). For example, some data identify that endothelial TLR4 critically regulates intestinal perfusion, leading to NEC, and reveal that the protective properties of breast milk involve enhanced intestinal microcirculatory integrity (284). Endothelial TLR4 activation impairs intestinal microcirculatory perfusion in necrotizing enterocolitis also via eNOS-NO-nitrite signaling (endothelial nitric oxide synthase). HMO-2'FL protects against NEC in part through maintaining mesenteric perfusion via increased eNOS expression (285).

*In vitro* and *ex vivo* animal studies have suggested an important role for milk-derived cytokines such as TNF (59), MFG-E8 (286), and IL-6 (96) on intestinal epithelial repair. Numerous functions in wound repair, coagulation, and host defense have been attributed to glycosaminoglycans (chondroitin sulfate, heparin sulfate, hyaluronan dermatan sulfate) (287), and many clinical studies have demonstrated the effectiveness of glycosaminoglycan preparations in enhancing wound healing (288).

Just about the reparative capacity, we recall the growth factors present in breast milk, as EGF promotes the growth and maturation of the fetal pulmonary epithelium and accelerates wound healing andVEGF, whose main role is the mediation of angiogenesis (27). Enteral EGF administration can increase the expression of the anti-apoptotic protein Bcl-2 and decrease levels of the pro-apoptotic protein Bax. The role of EGF in balancing apoptosis regulators provides implications of an opportunity for future therapeutic strategies to protect the intestinal barrier from injury in NEC (289, 290).

Growth factors are also secreted by breast milk stem cells (180). In fact, the concept for the potential therapeutic benefit of human milk stem cells (HMSCs) is largely based on the results of studies where HMSCs were cultured and differentiated *in vitro* followed by identification of the growth factors that they secreted (291, 292). For example, of particular importance for stroke therapy is the ability of certain subpopulations of these differentiated cells to produce VEGF and HGF and a protocol for culturing breast milk-derived cells that increase the production of these factors, which has been established (293). The regenerative power of HMSCs has not yet been defined, but the studies are very promising.

### Human Milk Opportunities

During this period, the scientists are studying the disease caused by SARS-CoV-2, how bioactive factors of milk can modulate vertical transmission or otherwise affect the clinical presentation/disease trajectories of newborns. Milk from mothers who have COVID-19 could be a source of antibodies directed against the virus. Data indicate that there is strong sIgA-dominant SARS-CoV-2 immune response in human milk after infection in the majority of individuals (294). As with plasma antibodies, these maternally derived antibodies offer the potential as a therapy to help seriously ill patients (242).

But, since the set of all the factors would seem more effective in protecting and combating the infection, Dutch researchers (295), instead of isolating antibodies, plan to use whole milk as therapy and are studying various pasteurization techniques that make milk safe but also retain all important immune components (SARS-CoV-2 Research Highlights the Importance of Human Milk Immunobiology. International Milk Genomics Consortium, July 2020).

### Multifunctionality

**Figure 5:**
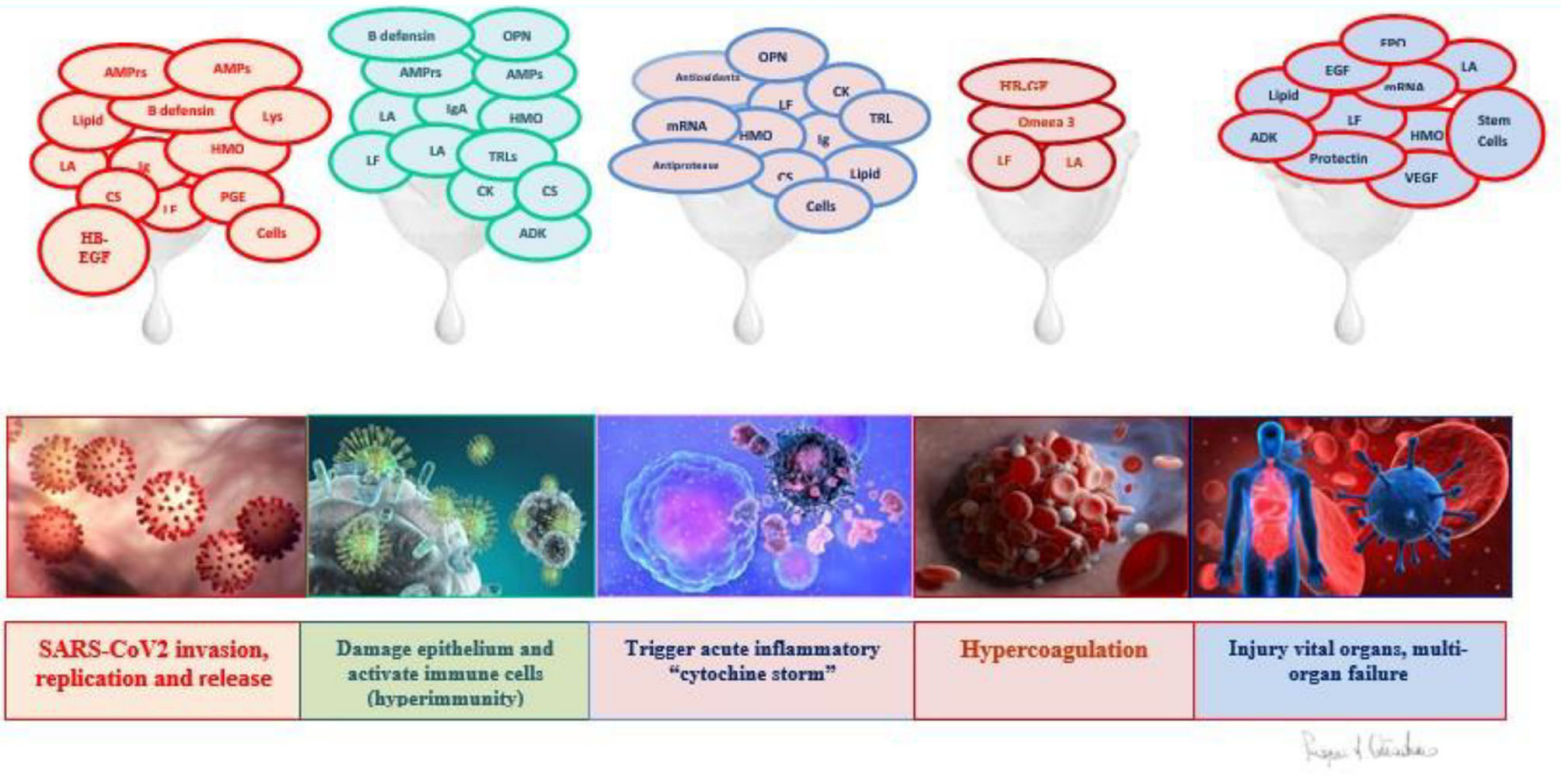
Functions of breast milk factors in pathogenetic events of COVID disease. LF, lactoferrin; LA, Lactadherin; CK, Cytokines; CS, Chondroitin Sulfate; HMO, Oligosaccharides; AMPrs, Antimicrobial Proteins; AMPs, Antimicrobial Peptides, OPN, Osteopontin; TGFβ, transforming growth factor; TNFα, Tumor Necrosis Factor; EGF, Epidermal Growth Factor, HB-EGF, Heparin Binding EGF-like growth factor, VEGF, Vascular Endothelial Growth Factor; EPO, Erythropoietin, Lipids: omega-3 PUFAs, Omega-3+omega-6 PUFAs, FFAs: Free Fatty Acids, ADK: Adipokines, Ig: SIgA: Secretory IgA, IgM, IgG, Lys: Lysozyme, B defensing, TLRs: Toll-Like Receptor, Antioxidants, Anti-proteases (α-1-antitrypsin, α-1-antichymotrypsin, elastase inhibitor), mRNA: MicroRNA, Cells: Lactobacillus Spp, Bifidobacterium Spp, Leukocytes, Stem cells, Protectin, PGE1/E2: Prostaglandin E1/E2.

Most of the protective components of human milk may interact synergistically with each other or with factors related to the mucosal or systemic immune response (99). Moreover, the main factors are multifunctional and exhibit both antimicrobial and anti-inflammatory activities but also control of immune hyperactivity and some have simultaneously shown repairing skills (Figure 5). As demonstrated in some studies, multiple compounds that act through different mechanisms can exert combined protective and curative effects, and future investigations should evaluate the application of therapies consisting of a set of multiple components from milk (Figure 3).

Furthermore, since the majority of bioactive factors in milk have not yet been identified, characterization of novel factors in milk will open the door to the development of novel antimicrobial therapies.

Giving seriously ill patients human milk would allow them to take advantage of the synergistic activities of milk immune factors that have evolved to improve survival in vulnerable infants and young children.

### Tolerability

Human milk factors are well-tolerated and practically free of side effects, and ongoing studies should be intensified while others should be activated (276, 277). Their efficiency should be the maximum expression of the antiviral, anti-inflammatory, and immune modulation functions during the complex course of COVID-19 or as protective factors for the entire population, especially that part which is at the greatest risk. A goal could be the discovery of factors capable to reduce the severe evolution of the disease and the use of respiratory assistance in intensive care.

### For the Infants

All the infants of the world must be fed with breast milk; in this way, they may be protected against COVID-19. The breastfeeding must extend over a period of 2 years (at least), as indicated by the WHO. Human milk with its mediators represents a complex immune element, which varies its composition to meet the needs of the mothers and children. Where mother's milk is not available, the alternative for premature baby or newborns with pathologies must be represented by human milk donated to the Human Milk Banks, which, although pasteurized, retains most of its anti-infectious properties (e.g., total savings of HMOs and fatty acids). Whenever human milk substitutes are needed, formulas added to milk bioactive factors should be preferred.

## Conclusions

In recent years, due to the significant number of viral disease outbreaks worldwide, vaccines and antiviral drugs to control the spread are unable to account for the mutation rates of viruses and are not developing fast enough compared to the exponential spread of the disease. Human milk with its bioactive factors offers a potent solution to prevent and fight life-threatening infections. Many data are available about the common viral infections; few data about MERS and CoV and emerging data about CoV-2 are published. Immunoglobulins, LF, and HMOs with many other active substances represent antiviral mediators against the new CoV-2 that underline the important role of breastfeeding and of human milk as a possible source of protective and therapeutic elements during this pandemic infection.

Some of the bioactive components of milk are undergoing preclinical evaluation in various disease models, others are subjected to active clinical evaluation, and others are in clinical use.

The puzzle of the COVID infection is being put together over this period of months; however, the knowledge of breast milk is a much larger and complex puzzle, which is missing several pieces, and that is why we talk about the “mystery of breast milk,” and who knows, possibly from the study of this mystery, answers can be gained to help with the great epidemics of humanity.

### Limitations

Only few data are available about the human milk's potential role against COVID-19, but a lot of studies documented the antiviral effect against many viruses.

The paper noted that some actions of breast milk factors were designed for use in adults, but most functions were described predominantly in the newborn.

The prevalent animal trial has been completed for other viral infections, and we need clinical trials for the better result.

## Data Availability Statement

The original contributions presented in the study are included in the article/supplementary materials, further inquiries can be directed to the corresponding author/s.

## Author Contributions

All authors contributed to the article and approved the submitted version.

## Conflict of Interest

The authors declare that the research was conducted in the absence of any commercial or financial relationships that could be construed as a potential conflict of interest.

## Abbreviations

SARS-CoV-2: severe acute respiratory syndrome-coronavirus 2
SIgA: secretory IgA
SIgM: secretory IgM
HMO: human milk oligosaccharides
20-FL: 20-fucosyllactose
30-SL: 30-sialyllactose
60-SL: 60-sialyllactose
LNnT: lacto-N-neotetraose
AIEC: adherent-invasive *Escherichia coli*
LNT: lacto-N-tetraose
SCFA: short-chain fatty acid
CD4+ and CD8: T lymphocytes
Treg: regulatory T cells
NF-κB: nuclear factor kappa Beta
TLR: toll-like receptor
IL: Interleukin
PAMP: pathogen-associated molecular pathways
PNC: platelet–neutrophil complex
IFNγ: interferon γ
TNF: tumor necrosis factor
TGF: transforming growth factor
G-CSF: granulocyte colony-stimulating factor
M-CS: macrophage colony-stimulating factor
GM-CSF: granulocyte macrophage colony-stimulating factor
IL-1RA: IL-1 receptor antagonist
NEC: necrotizing enterocolitis
Th1: T helper type 1
Th2: T helper type 2
NK: natural killer
TGF-β: transforming growth factor β
FOXP3: forkhead box P3
EGF: epidermal growth factor
HB-EGF: heparin-binding epidermal growth factor-like growth factor
VEGF: vascular endothelial growth factor
CXC: chemokines
SFA: saturated fatty acid
MUFA: monounsaturated fatty acid
PUFA: polyunsaturated fatty acid
PPAR: peroxisome proliferator-activated receptor
PAF: platelet-activating factor
COX-2: cyclooxygenase 2
SPM: specialized pro-resolving mediator
FFA: free fatty acid
pIgR: polymeric immunoglobulin receptor
LF: lactoferrin
LBP: lipopolysaccharide binding protein
ICAM-1: intercellular adhesion molecule 1
CD54: cluster of differentiation 54
HBD: human β-defensin
OPN: osteopontin
ROP: retinopathy of prematurity
ROS: reactive oxygen species
SOD: superoxide dismutase
GPx: glutathione peroxidase
miRNA: microRNA
DC: dendritic cell
RSV: respiratory syncytial virus
HBGA: histo-blood group antigens
PGE: prostaglandins
MERS: Middle East respiratory syndrome
IUGR: intrauterine growth restriction
WHO: World Health Organization
CDC: Centers for Disease Control and Prevention
AAP: American Academy of Pediatrics
ACE-2: angiotensin-converting enzyme 2
RBD: receptor-binding domain
TMPRSS: type II transmembrane serine protease
LNFP III: lacto-*N*-fucopentaose III
sTLR: soluble Toll-like receptor
sCD: soluble cluster of differentiation
MFG-E8: milk fat globule-epidermal growth factor-factor VIII.
